# Spina Ventosa of the Right Second Metacarpal in an Indian Girl With No Pulmonary Involvement: A Rare Case

**DOI:** 10.7759/cureus.117263

**Published:** 2026-09-30

**Authors:** Sankalp Yadav, Amruta Nadar, Madhan Jeyaraman, Gautam Rawal, Naveen Jeyaraman

**Affiliations:** 1 Medicine, Shri Madan Lal Khurana Chest Clinic, New Delhi, IND; 2 Medicine, Ayushman Arogya Mandir Subcenter, New Delhi, IND; 3 Regenerative Medicine, Agathisha Institute of Stemcell and Regenerative Medicine (AISRM), Chennai, IND; 4 Orthopaedics, ACS Medical College and Hospital, Dr. M.G.R. Educational and Research Institute, Chennai, IND; 5 Respiratory Medical Critical Care, Max Super Speciality Hospital, New Delhi, IND

**Keywords:** antituberculous treatment, cartridge based nucleic acid amplification test (cbnaat), mycobacterium tuberculosis (mtb), second metacarpal, tb – tuberculosis

## Abstract

Tuberculosis (TB) of the small bones of the hand is rare, especially in children. Isolated metacarpal involvement is even less common. We report a rare case of isolated TB of the second metacarpal in a five-year-old Indian girl without pulmonary or systemic involvement. She presented with localized pain and swelling over the dorsum of the hand. The diagnosis was challenging because of nonspecific clinical features and the absence of constitutional symptoms. Cartridge-based nucleic acid amplification testing confirmed *Mycobacterium tuberculosis*, allowing timely initiation of weight-based antituberculous therapy. This case emphasizes the need to consider skeletal TB in children with persistent, unexplained hand swelling, even without evidence of pulmonary disease.

## Introduction

Tuberculosis (TB), caused by *Mycobacterium tuberculosis*, remains a major cause of morbidity and mortality worldwide despite advances in diagnosis and treatment [1]. Although pulmonary disease predominates, extrapulmonary TB accounts for approximately 10%-15% of cases and may involve the musculoskeletal system [2]. Skeletal TB is uncommon in children and is thought to result primarily from lymphohematogenous dissemination during primary infection [3].

Tuberculous involvement of the small bones of the hand and foot, encompassing the metacarpals, metatarsals, and phalanges, is referred to as tuberculous dactylitis or spina ventosa [4,5]. It predominantly affects young children, with approximately 85% of cases occurring before six years of age [6].

Isolated TB of a single metacarpal is particularly rare and may mimic other infective, inflammatory, or neoplastic conditions. Recognition of this unusual presentation is therefore essential, especially in children from TB-endemic regions. Herein, we report a rare case of isolated TB of the second metacarpal in a five-year-old girl without pulmonary involvement, in whom molecular confirmation of *M. tuberculosis* established the diagnosis and facilitated appropriate treatment.

## Case presentation

In the fall of 2024, a five-year-old girl was brought to the outpatient department by her father with complaints of dull, aching pain and swelling over the dorsum of her right hand for two months. There was no erythema or discharging sinus. There was no history of trauma, TB exposure, sick contacts, or constitutional symptoms suggestive of TB. She lived in a slum and had no history of stays in a refugee camp or prison. She was fully vaccinated as per her age.

General examination revealed a young, hemodynamically stable girl with an ectomorphic habitus. There was no pallor, icterus, cyanosis, clubbing, or pedal edema. Systemic examination was unremarkable.

Local examination revealed a localized swelling 5 cm x 3.5 cm over the dorsal aspect of the right hand, centered at the base of the metacarpal. It was tender to touch, and the local temperature was slightly raised. There was no overlying erythema or discharging sinus. The range of motion at the metacarpophalangeal and proximal interphalangeal joints was restricted (Figure 1).

**Figure 1:**
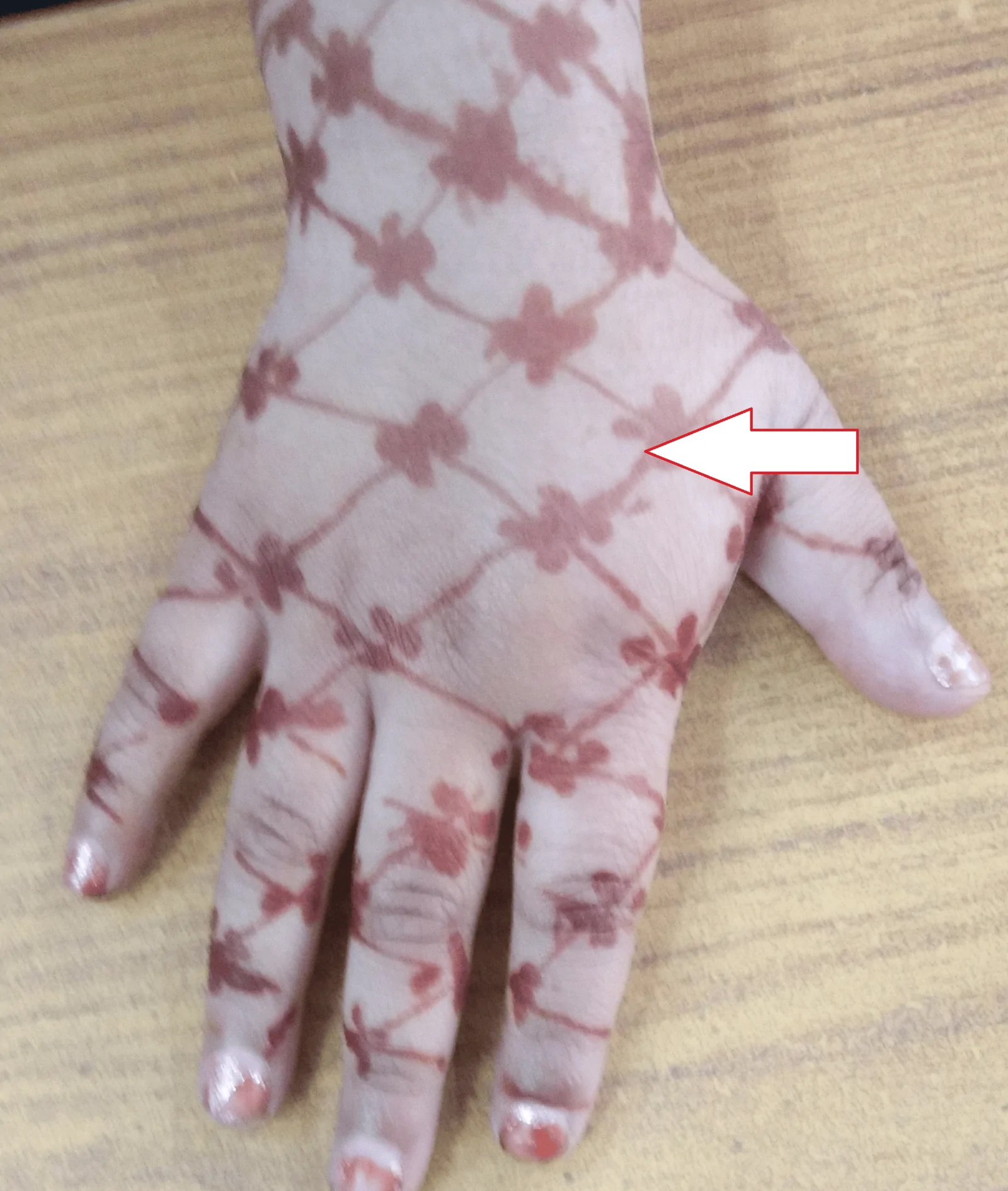
Localized swelling over the dorsal aspect of the right hand

The differential diagnoses included TB, aneurysmal bone cyst, foreign body granuloma, osteoblastoma, syphilitic, and fungal dactylitis; however, persistent osteomyelitis was initially considered. Laboratory evaluation revealed elevated C-reactive protein and erythrocyte sedimentation rate, with a strongly positive Mantoux test. Details are mentioned in Table 1.

**Table 1:** Laboratory data HIV: Human immunodeficiency virus; VDRL: Venereal disease research laboratory.

Test	Result	Normal range
C-reactive protein	60 mg/L	< 10 mg/L
Erythrocyte sedimentation rate	84 mm/h	0 to 10 mm/h
Mantoux test	20 × 18 mm	<10 mm
HIV	Non-reactive	Non-reactive
Hepatitis A, B, C, D, E	Negative	Negative
Rheumatoid factor	Negative	Negative
VDRL	Negative	Negative
Induced sputum smear microscopy	Negative	Negative

A chest radiograph showed no evidence of pulmonary disease. A radiograph of the hand was suggestive of an expansile, geographic osteolytic lesion involving the shaft of the second metacarpal of the right hand with cortical thinning and mild osseous expansion, giving a cystic/spina-ventosa appearance (Figure 2).

**Figure 2:**
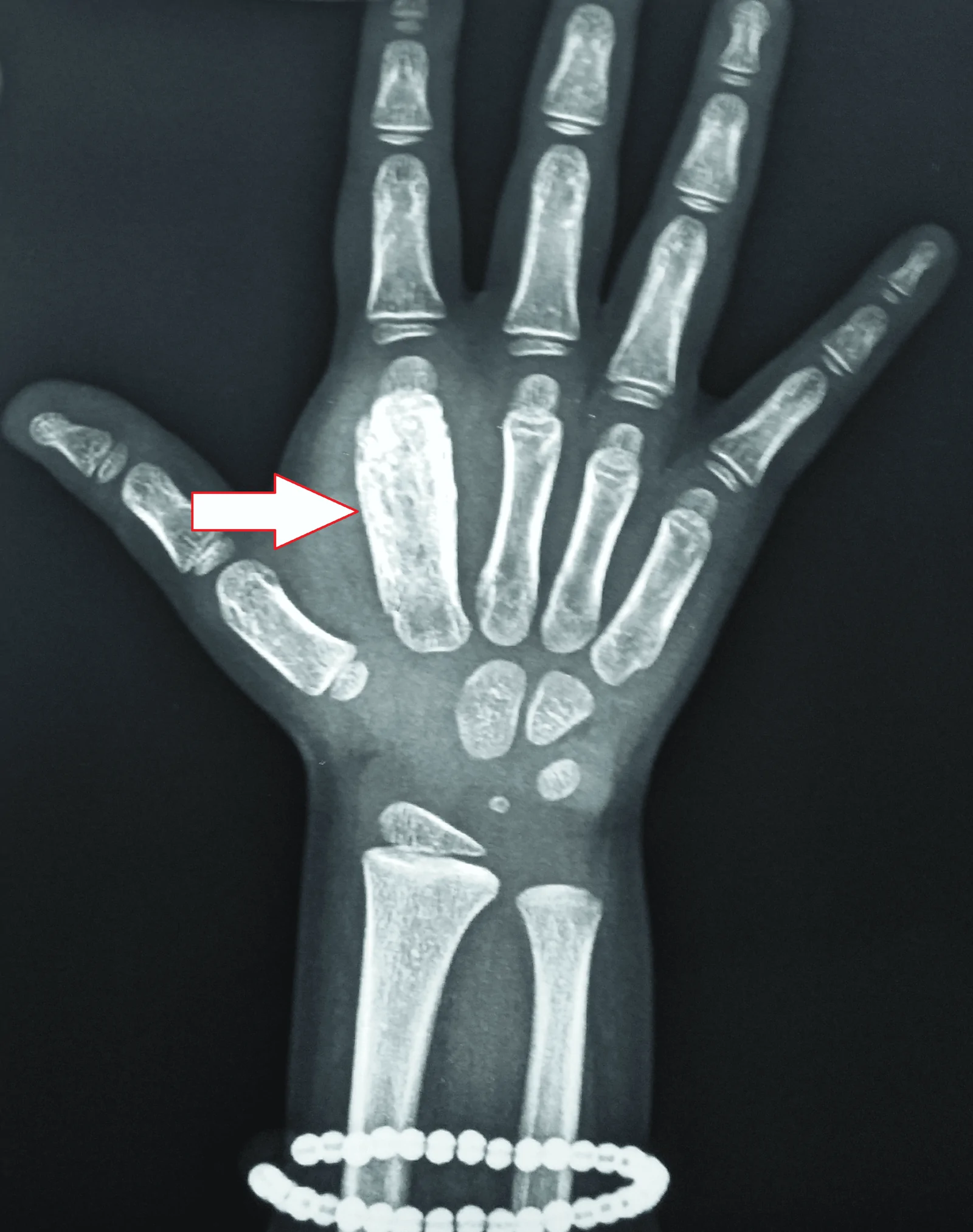
A plain radiograph of the right hand showing an expansile, geographic osteolytic lesion involving the second metacarpal

MRI of the hand was offered but not performed, as the patient's parent refused it due to financial constraints. Ultrasound-guided bone biopsy revealed granulomatous inflammation predominantly comprising lymphocytes, with occasional Langhans giant cells and areas of necrosis. Samples were subjected to Gram staining and bacterial, fungal, and mycobacterial cultures, all of which were negative. However, cartridge-based nucleic acid amplification testing (CBNAAT) detected *M. tuberculosis* at a very low level, with no evidence of rifampicin resistance.

A final diagnosis of spina ventosa involving the right second metacarpal, without pulmonary involvement, was established. Antituberculous therapy was initiated with a pediatric fixed-dose combination of isoniazid, rifampicin, ethambutol, and pyrazinamide for two months, followed by isoniazid, rifampicin, and ethambutol for an additional 10 months.

The patient showed clinical improvement with medical management, with a progressive reduction in swelling and pain and no significant adverse drug reactions. After completing four months of antituberculous therapy, her parents requested transfer to their village for continuation of care, which was facilitated. Her treatment outcome was subsequently recorded as “treatment completed” in the national TB surveillance portal, Nikshay.

## Discussion

TB remains highly prevalent in low- and middle-income resource-limited settings. Extrapulmonary TB is relatively infrequently reported, while musculoskeletal involvement is reported in only 1%-3% of patients and constitutes 15% of extrapulmonary disease [7].

TB of the small bones of the hand is particularly uncommon, with limited published literature even from TB-endemic regions. Further, TB of the metacarpals constitutes one percent of all skeletal TB cases [8]. This rarity, coupled with its often nonspecific clinical and radiological presentation, can make diagnosis challenging and contribute to delays in treatment.

Spina ventosa, or tuberculous dactylitis, was first described by Boyer in 1803, with its tuberculous etiology subsequently established by Nelaton in 1837. Histological and radiographic descriptions followed in the late nineteenth century [5]. The term describes tuberculous involvement of the small bones, characterized by medullary destruction, cortical thinning, and fusiform expansion. It accounts for approximately 2%-4% of skeletal TB cases and most commonly affects the phalanges, particularly those of the index and middle fingers [4]. Because of its indolent course, paucibacillary nature, and infrequent systemic manifestations, tuberculous dactylitis remains uncommon and often underrecognized [5].

The condition is frequently characterized by localized pain and swelling, while constitutional and pulmonary manifestations may be absent. Early radiographs can be nonspecific, and the paucibacillary nature of skeletal TB further complicates microbiological confirmation, potentially leading to delayed diagnosis. Additional challenges include limited clinical suspicion, overlap with other musculoskeletal disorders, and nonspecific multimodal imaging findings [9].

Early imaging may show only soft-tissue swelling and periostitis, which can progressively evolve into expansile osteolysis, cortical destruction, and sequestrum formation [10]. These features underscore the importance of maintaining a high index of suspicion and pursuing microbiological confirmation in children with persistent or unexplained digital or metacarpal lesions [5].

Antituberculous therapy remains the mainstay of treatment for tuberculous dactylitis [4,5]. Treatment is generally effective, with a duration of approximately 12 months, followed by clinical and radiological reassessment to determine the need for further management [5,9].

A case similar to ours was detailed by Aghoutane et al. (2017), in which a three-year-old girl was diagnosed with spina ventosa of the fifth metacarpal [9]. Our case shares similarities, with no history of trauma or pulmonary involvement, a raised erythrocyte sedimentation rate, a positive Mantoux test, and medical management without surgical intervention. However, the presented case differs in early diagnosis, raised C-reactive protein levels, and detection of *M. tuberculosis* on CBNAAT.

## Conclusions

This case highlights that isolated tuberculous dactylitis or spina ventosa can occur in otherwise healthy children without pulmonary disease or constitutional symptoms. It may also occur without trauma or a previous history of TB. Skeletal TB should therefore be considered in children with persistent digital swelling or pain, particularly in endemic regions. Microbiological and histopathological confirmation is important because clinical and radiological findings may be nonspecific. Early diagnosis and appropriate antituberculous therapy can lead to good outcomes and avoid unnecessary delays or interventions.
